# A Rare Case of a Nasopharyngeal Pleomorphic Adenoma Mimicking a Nasopharyngeal Carcinoma

**DOI:** 10.7759/cureus.31269

**Published:** 2022-11-08

**Authors:** Siti Farhana Abdul Razak, Mohd Aizat Abdul Aziz, Aneeza Khairiyah Wan Hamizan, Elsie Jane Anastasius

**Affiliations:** 1 Otolaryngology - Head and Neck Surgery, Hospital Queen Elizabeth, Kota Kinabalu, MYS; 2 Pathology, Hospital Queen Elizabeth, Kota Kinabalu, MYS; 3 Otolaryngology - Head and Neck Surgery, Universiti Kebangsaan Malaysia Medical Centre, Kuala Lumpur, MYS

**Keywords:** pleomorphic adenoma, nasopharyngeal carcinoma, nasopharynx, minor salivary gland tumour, nasopharyngeal pleomorphic adenoma

## Abstract

Pleomorphic adenoma is the most common benign pathology of the major salivary gland but rare in the minor salivary gland, especially in the nasopharynx, with only a few cases reported in the literature. A 76-year-old lady presented with bilateral nasal blockage for one year secondary to a nasopharyngeal mass. Histopathological examination reported it to be nasopharyngeal carcinoma, but the mass persisted after a course of chemotherapy combined with radiotherapy. Upon repeat biopsy, the mass was found to be a pleomorphic adenoma. The patient underwent nasopharyngectomy without complications and no evidence of recurrence after 18 months of follow-up.

## Introduction

Pleomorphic adenoma is the most common benign neoplasm seen in the major salivary glands, predominantly seen in the parotid gland. However, it is also seen along the minor salivary gland located in the upper aerodigestive tract, with the palate as the most common site seen. Pleomorphic adenoma of the nasopharynx is a rare occurrence as there are only a few cases reported in the literature [1]. Pleomorphic adenoma has components of both epithelial and mesenchymal in origin, therefore often dubbed as ‘mixed tumors’. Although benign, this pathology can be locally invasive with a risk of malignant transformation [2]. The presenting symptoms may vary between patients as it is associated with the size and site of the tumor. Surgical resection is the mainstay treatment modality. Here. we report a case of a nasopharyngeal pleomorphic adenoma that was successfully resected using an endoscopic transnasal approach.

## Case presentation

**Figure 1:**
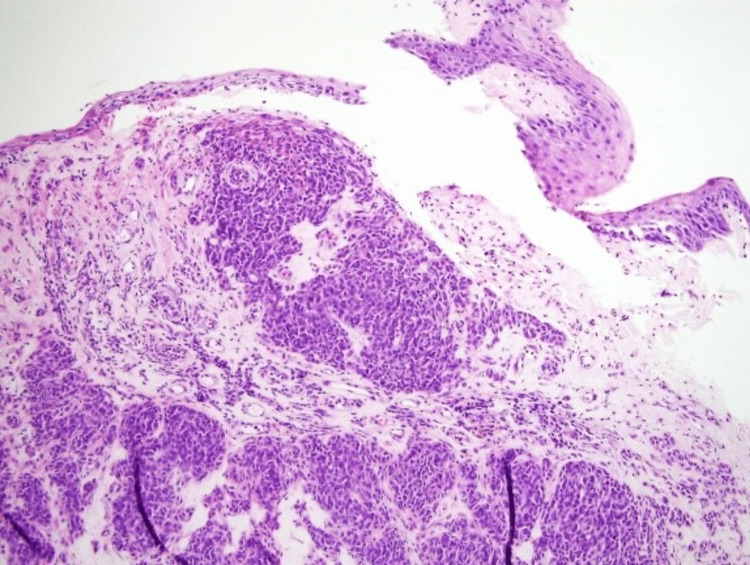
Fragments of tissue lined by benign stratified squamous epithelium (x100 magnification, hematoxylin and eosin stain). The underlying stroma is infiltrated by malignant cells arranged in sheets and trabeculae pattern

A 72-year-old native lady presented to a district hospital with the chief complaint of chronic bilateral nasal blockage for the past one year, associated with a bilateral reduction in hearing and intermittent epistaxis. She has no family history of nasopharyngeal carcinoma and no environmental factor that was predisposed to nasopharyngeal carcinoma. Nasoendoscopy showed a smooth mass, covered with slough from the right fossa of Rosenmuller (FOR), which had extended to the posterior choana; there was no clinically palpable cervical lymph node and all cranial nerves were intact. Biopsy for histopathological examination was reported as nasopharyngeal carcinoma (Figure 1) with positive CK5/6, and no Epstein-Barr encoding region in situ hybridization (EBER ISH) staining was performed for this patient. No Epstein-Barr virus (EBV) DNA was taken for this patient. Computed tomography (CT) scan from the base of the skull to the pelvis revealed an enhancing mass arising from the right FOR (4.0 x 3.1 x 2.7 cm) extending anteriorly and crosses the posterior choana and posterior margin of the right middle turbinate, superiorly it abuts the clivus and base of sphenoid sinus and posteriorly, it had a close proximity with prevertebral muscle. There were no regional lymphadenopathy and no distant metastasis. The patient was diagnosed with nasopharyngeal carcinoma T1N0M0, based on American Joint Committee on Cancer, eighth edition, and referred to the oncologist for further treatment.

**Figure 2:**
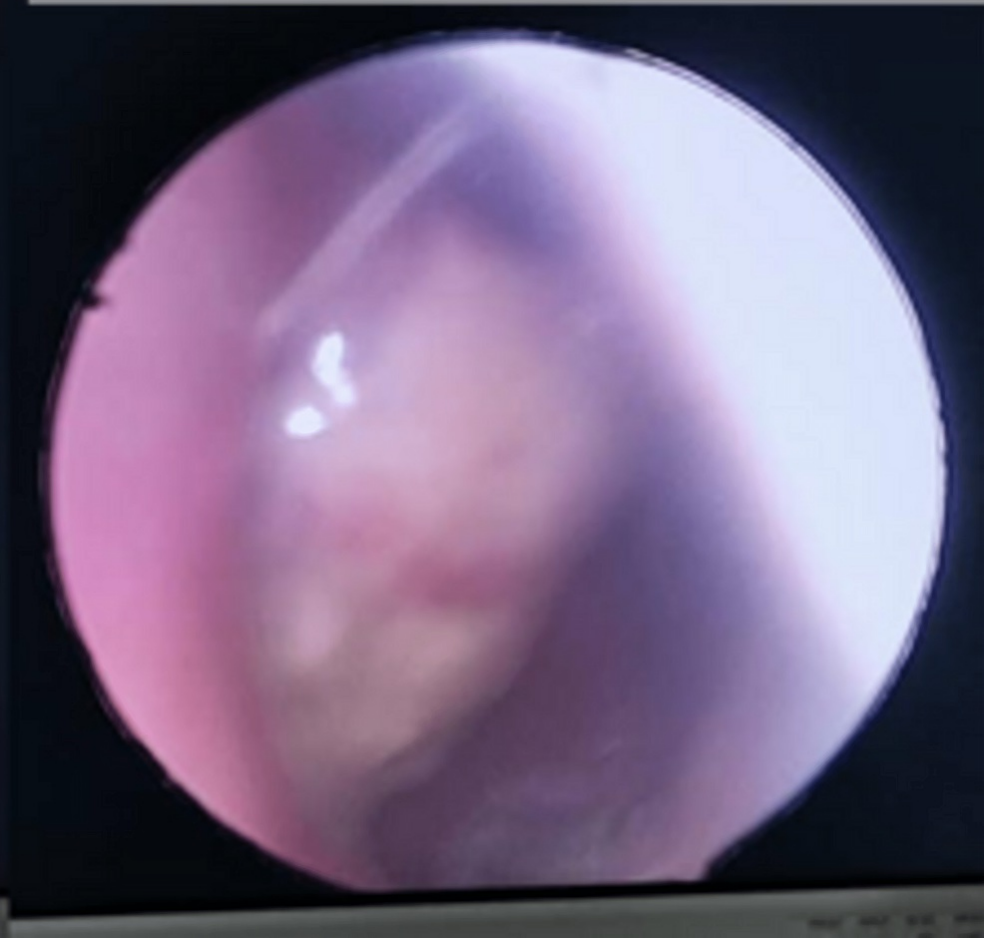
Nasoendoscopy on follow-up one month after radiotherapy showed a sloughy mass arising from the right FOR occupying the nasopharynx and extending anteriorly to the posterior choana FOR: fossa of Rosenmuller

**Figure 3:**
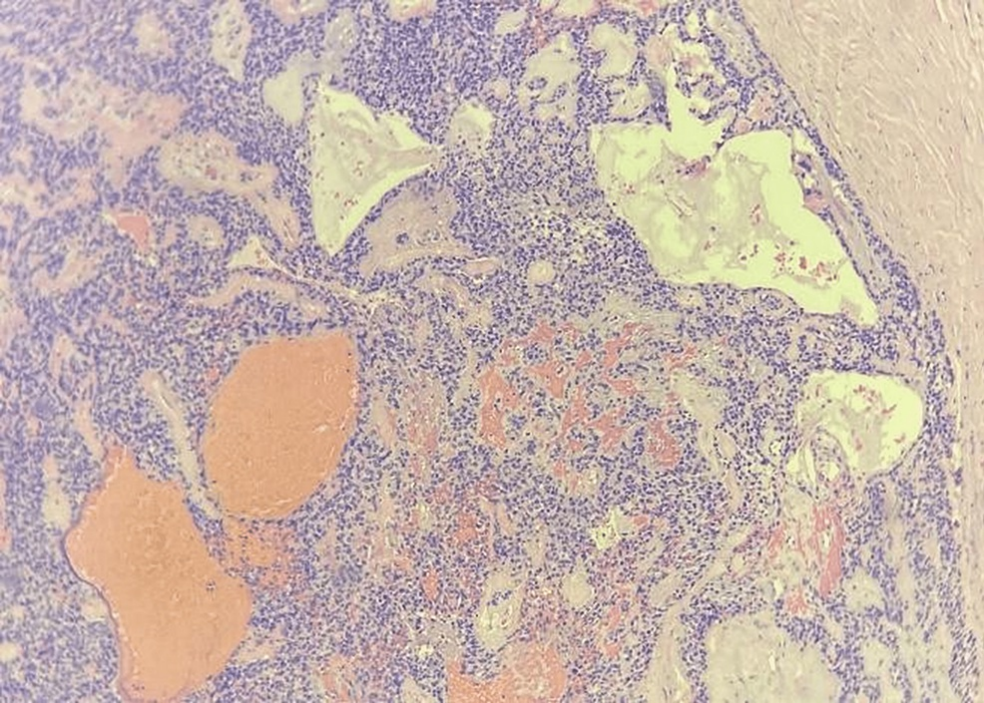
Neoplastic epithelial cells arranged in sheets and trabeculae with some ductal structures (x10 magnification)

**Figure 4:**
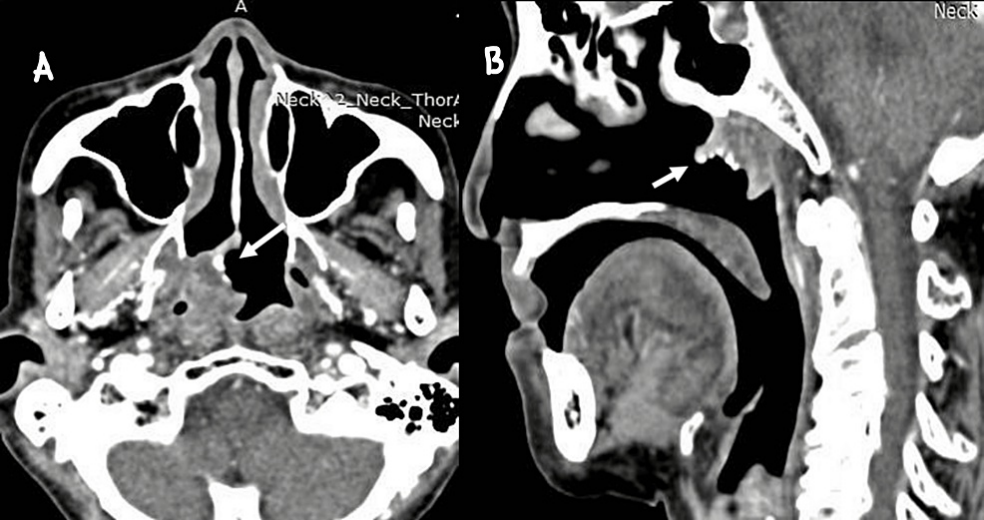
(A) Axial cut of CT scan showed tumour arising from the right nasopharynx with calcification within it (white arrow); (B) Sagittal cut of CT scan showed irregular tumour at the roof of nasopharynx with calcification within it (white arrow)

The patient completed radiotherapy with a total radiation dose of 70 Gy in 35 fractions. Upon follow-up one month after completion of radiotherapy, the mass in the right FOR was slightly smaller but had a similar extension into the right posterior choana (Figure 2). A repeat biopsy revealed unencapsulated salivary neoplastic abluminal cells with positive p63 and CK 5/6 stains, which were highly suggestive of pleomorphic adenoma (Figure 3). CT scans three months after the completion of radiotherapy reported that the right FOR mass was smaller (2.0 x 3.8 x 3.4 cm) without any regional or distant metastasis (Figure 4).

**Figure 5:**
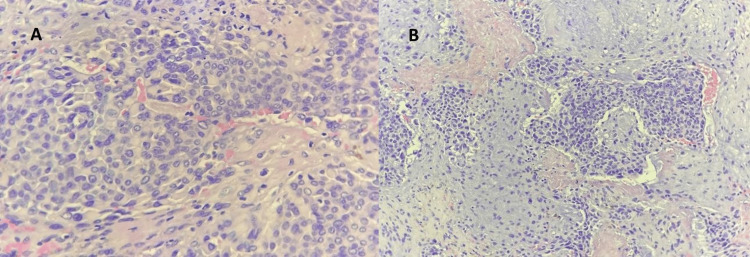
(A) Epithelial cells cuboidal to spindle in shape; (B) Intervening stroma appeared chondromyxoid Under x100 magnification

The patient then underwent endoscopic nasopharyngectomy under general anaesthesia. Intraoperatively, a lobulated, smooth surface tumour was seen arising from the right FOR where it crosses the midline and extends anteriorly to the posterior choana, superiorly approaching the roof of the nasopharynx; laterally it abuts the right torus tubarius and posteriorly the prevertebral fascia appeared not involved. The tumour was removed using a microdebrider until superiorly to the roof of the nasopharynx, inferiorly to the Passavant ridge, laterally at the torus bilaterally, and posteriorly until the prevertebral fascia, since it was done endoscopically. Histopathology of the nasopharynx showed neoplastic epithelial cells that were arranged in sheets and trabeculae, with some ductal structures. The ducts consisted of luminal epithelial and abluminal myoepithelial cells. The intervening stroma appeared myxoid with chondroid differentiation. Occasional mitoses were similarly seen in the nasopharynx specimen. There was an absence of nuclear atypia, atypical mitosis, and perineural or lymphovascular invasion; hence, no evidence of malignancy was observed (Figure 5). The patient was discharged home two days after the operation and subsequent follow-ups in the clinic showed no complications and no evidence of recurrence.

## Discussion

Nasopharyngeal carcinoma is a neoplasm that arises from the epithelial cells of the nasopharynx. It is the fifth most common malignancy in Malaysia according to Global Cancer Observatory 2020 (Globocan) Malaysia [3]. Minor salivary gland tumour of the nasopharynx is rare. Only 20% of all pleomorphic adenoma originates from the minor salivary gland and among them, pleomorphic adenoma of the nasopharynx is uncommon [4]. Previously, there were 11 cases of nasopharyngeal pleomorphic adenoma reported in a study by Bowman et al. and one patient reported by Karligkiotis et al., making a total of 12 cases seen in the literature [2,5]. Pleomorphic adenoma might be mistaken as a malignant neoplasm when it grows at unusual anatomical sites, such as the nasopharynx in this case. Garcia's study reported a case of nasopharyngeal carcinoma T2N0 patient who had completed radiotherapy and presented with unilateral ear obstruction and a rounded mass arising from the right wall of the nasopharynx during nasoendoscopy [4]. The patient was arranged for operation and an intraoperatively frozen section was reported as well-differentiated squamous cell carcinoma; however, the final diagnosis based on a cytopathologic study was consistent with pleomorphic adenoma. Thakur et al. treated a patient based on fine needle aspiration cytology performed on a huge intraoral mass, which was reported as possible squamous cell carcinoma [6]. Despite chemo-radiation, the tumour did not regress; thus, an intraoral biopsy was done, which was highly suggestive of pleomorphic adenoma. Clinicians need to always consider other differentials and communicate with the pathologist when dealing with an atypical presentation of nasopharyngeal carcinoma.

Typically, nasopharyngeal carcinoma presents with painless cervical lymphadenopathy, nasal obstruction, epistaxis, halitosis, ear blockage, tinnitus, otalgia, diplopia, ptosis, trismus, dysphagia, and hoarseness of voice The incidence of nasopharyngeal carcinoma in Southeast Asia and Southern China is higher than the rest of the world. In Malaysia, it is commonly seen in the Chinese population, followed by the natives of Sabah and Sarawak, and seen mostly in the age group of 40-60 years [7]. Meanwhile, symptoms of pleomorphic adenoma vary in the literature; for example, epistaxis, nasal obstruction, otalgia, aural obstruction, hearing loss, and dysphagia [8]. The patient in the current report presented with nasal obstruction and hearing impairment, which were the common symptoms reported in both nasopharyngeal carcinoma and nasopharyngeal pleomorphic adenoma. In view of the patient’s age, being a Sabah native, and the high prevalence of nasopharyngeal carcinoma in Southeast Asia, it was the most likely diagnosis for this patient upon presentation. On the contrary, pleomorphic adenoma of the minor salivary glands has a female preponderance and was common in patients between 30 to 60 years of age [1]. There was no racial predilection reported for nasopharyngeal pleomorphic adenoma as yet. Clinical diagnosis of nasopharyngeal pleomorphic adenoma was difficult to be made as similar symptoms might be observed in nasopharyngeal carcinoma. In retrospect, the lack of neck node involvement may have been an indicator that this is unlikely to be nasopharyngeal carcinoma.

Nasoendoscopy had a role in detecting nasopharyngeal mass and aiding in a sampling of nasopharyngeal biopsy. The tumour might appear differently, for example, the appearance of slight fullness or bulging in the FOR and contact bleed [9]. In advanced stages of nasopharyngeal carcinoma, a patient might have cranial nerve III, IV, V, VI palsy and cervical lymphadenopathy. Pleomorphic adenoma of the nasopharynx appeared as a rubbery, smooth surface mass without contact bleed and without surrounding structure destruction [10], and was covered with normal mucosa normally. Ulceration might be seen if there was an associated trauma or infection to the tumour [11]. This patient most likely had a benign neoplasm that pointed towards nasopharyngeal pleomorphic adenoma, given the clinical appearance of a smooth mass covered with ulceration with no contact bleeding, which was similar to the clinical appearance of the tumour described by Roh et al. [10].

The macroscopic appearance of nasopharyngeal carcinoma varies including smooth mucosal bulge, raised nodule with or without ulceration, and infiltrative lesion seen under the microscope. The microscopic appearance of nasopharyngeal carcinoma differs according to its subtype; for example, there will be squamous differentiation and keratinisation with a desmoplasmic response in the keratinising subtype of the nasopharyngeal carcinoma [12]. Macroscopically, pleomorphic adenoma has a capsule but it is not a true capsule as it is made of the salivary gland tissue that has been arranged to look like a capsule. It also has a pseudopod extension into the normal structure [13]. Microscopically, the presence of epithelial, myoepithelial, and stromal elements can denote the tumour as a pleomorphic adenoma, as evidenced by the patient’s nasopharynx specimen [14]. The first biopsy of this patient had stromal invasion, necrosis, apoptosis, and there was no lymphovascular or perineural invasion. However, CK5/6 was positive, and no EBER ISH was performed for this patient. It is possible that the first biopsy was a malignant salivary gland tumour as it had stromal invasion; however, the resected nasopharynx did not have nuclear atypia and no perineural or lymphovascular infiltration, which points it as a benign neoplasm. Due to the first biopsy being small, it might not have detected the presence of any duct components. Pleomorphic adenoma of the upper aerodigestive tract including the nasopharynx may mimic epithelial tumours due to its high cellularity combined with the lack of stromal components. Epithelial cells may also almost completely constitute the morphology, with a few or no stromal. This may contribute to the misdiagnosis of the disease, as presented in this patient [15].

Imaging plays an important role in the diagnostic workup of tumours located in the nasopharynx as it provides the key for the management of the next step. CT scan can provide an assessment for features of malignancy, the local extent of the tumour, and possible regional metastasis. CT scan of nasopharyngeal carcinoma typically showed contrast-enhanced soft tissue tumour with heterogenous enhancement. The tumour is most centred at the Rossenmuller fossa where a small tumour is confined to the nasopharynx and a large tumour may extend multi-directionally. Bony erosions were also observed in the case of large nasopharyngeal carcinoma [16]. CT scan of this patient showed a mass originating from right FOR with foci of calcification seen within the tumour as well as the presence of contrast enhancement. There was no bony destruction, no regional lymphadenopathy, and no distant metastasis seen in the CT scan, which is highly suggestive of a benign neoplasm. Pleomorphic adenoma typically appears as a well-defined mass of soft tissue density with a contrast enhancement [17], as seen in this patient. Martínez-Capoccioni et al. suggest that radiological imaging could not differentiate between low-grade malignancy and other benign tumours from time to time and, therefore, clinical findings should support the radiological findings [18].

Radiation therapy is the main treatment modality for primary non-disseminated nasopharyngeal carcinoma as the cancer itself is radiosensitive. The recommended regime is 66 to 70Gy in 33 to 35 fractions, treated once per day over six to seven weeks of duration. The addition of chemotherapy as an adjunct to radiotherapy in locoregional advance nasopharyngeal carcinoma is proven to improve the five-year survival rate for stages three and four of the disease. This patient was given radiotherapy with a dose of 70 Gy for 35 fractions, as per the clinical practice guidelines for the management of nasopharyngeal carcinoma [7]. However, one month post the completion of radiotherapy showed a persistent nasopharyngeal mass, thus raising suspicions of the possibility of other nasopharyngeal pathology.

In a retrospective analysis by Wallace et al. of 25 patients with pleomorphic adenoma treated with definitive radiotherapy, seven patients had tumour recurrence [19]. Two out of the seven patients underwent salvage surgery and were tumour free, one patient failed salvage surgery, but the author did not mention the contributing factor of failed surgery; one patient refused salvage surgery and another patient with unknown salvage surgery status. Adjuvant radiotherapy may be beneficial in patients with positive tumour margin, gross residual disease and recurrent multifocal disease as proven by various retrospective studies and it may reduce local recurrence rate as well [19-20]. Surgical excision is the treatment of choice in nasopharyngeal pleomorphic adenoma due to its nature that may progress in size if left untreated. The majority of documented cases followed the endoscopic transnasal approach as this method is associated with less morbidity, for example, facial deformity, nasal skeletal disruption, dental malocclusion, and iatrogenic eustachian tube injury [2]. The endoscopic method provides an excellent view of the surgical field and avoids destruction to the adjacent structures, especially the eustachian tube; thus, this approach is preferred compared to the open method [14]. The endoscopic approach was the preferred approach for the patient in the current case report to avoid all the complications listed above. No intraoperative complication was seen in the patient and no tumour recurrence was noted during the clinic review. Although no intraoperative frozen section was taken from this patient, it is recommended to take a frozen section specimen as it has a high accuracy rate between 88-98%; therefore, it can help to determine margin clearance and to differentiate between malignant or benign pathology of the salivary gland. Postoperative radiotherapy is usually not recommended in negative margin benign tumours as 95% achieve local control with surgical excision. Moreover, there is only minimal benefit of local control when given postoperative radiotherapy and there is an increased risk of radiation morbidity [20]. Therefore, surgical excision with a negative margin without radiotherapy is sufficient to treat nasopharyngeal pleomorphic adenoma.

Not all nasopharyngeal tumours are nasopharyngeal carcinoma; however, it is natural to think of nasopharyngeal carcinoma when a patient presented with a nasopharyngeal mass as it is the commonest tumour located in the nasopharynx. There are a few points that may suggest benign nasopharyngeal mass instead of nasopharyngeal carcinoma, for example, sociodemographic data including age, ethnicity, gender, lack of constitutional symptoms, negative family history of nasopharyngeal carcinoma, negative EBV from serology test, lack of cervical lymph nodes, and a characteristic smooth mass with no contact bleed. Therefore, a thorough history should be taken, and a detailed examination should be performed on all patients presented with nasopharyngeal mass. It is recommended to proceed with magnetic resonance imaging when there is suspicion of the clinical diagnosis as it provides a better soft tissue image compared to a CT scan. When there is a huge nasopharyngeal mass, for example as seen in this case, a large biopsy should be taken at multiple sites of the tumour as it will increase the likelihood of getting other microscopic features such as myoepithelial and duct components, which are commonly seen in pleomorphic adenoma.

## Conclusions

Nasopharyngeal pleomorphic adenoma is a rare tumour of minor salivary glands. It may mimic nasopharyngeal carcinoma as it may have a similar clinical presenting symptoms and as the upper aerodigestive tract have a higher epithelial composition with minimal or low stromal as compared to other major salivary glands. Endoscopic excision of the tumour is the mainstay of treatment for nasopharyngeal carcinoma as it provides a better visual field and may avoid complications related to open approach.
